# Disruption of the Molecular Regulation of Mitochondrial Metabolism in Airway and Lung Epithelial Cells by Cigarette Smoke: Are Aldehydes the Culprit?

**DOI:** 10.3390/cells12020299

**Published:** 2023-01-12

**Authors:** Christy B. M. Tulen, Antoon Opperhuizen, Frederik-Jan van Schooten, Alexander H. V. Remels

**Affiliations:** 1 Department of Pharmacology and Toxicology, School of Nutrition and Translational Research in Metabolism (NUTRIM), Maastricht University Medical Center+, P.O. Box 616, 6200 MD Maastricht, The Netherlands; 2 Office of Risk Assessment and Research, Netherlands Food and Consumer Product Safety Authority, P.O. Box 43006, 3540 AA Utrecht, The Netherlands

**Keywords:** aldehydes, cigarette smoke, chronic obstructive pulmonary disease, mitochondria, lung

## Abstract

Chronic obstructive pulmonary disease (COPD) is a devastating lung disease for which cigarette smoking is the main risk factor. Acetaldehyde, acrolein, and formaldehyde are short-chain aldehydes known to be formed during pyrolysis and combustion of tobacco and have been linked to respiratory toxicity. Mitochondrial dysfunction is suggested to be mechanistically and causally involved in the pathogenesis of smoking-associated lung diseases such as COPD. Cigarette smoke (CS) has been shown to impair the molecular regulation of mitochondrial metabolism and content in epithelial cells of the airways and lungs. Although it is unknown which specific chemicals present in CS are responsible for this, it has been suggested that aldehydes may be involved. Therefore, it has been proposed by the World Health Organization to regulate aldehydes in commercially-available cigarettes. In this review, we comprehensively describe and discuss the impact of acetaldehyde, acrolein, and formaldehyde on mitochondrial function and content and the molecular pathways controlling this (biogenesis versus mitophagy) in epithelial cells of the airways and lungs. In addition, potential therapeutic applications targeting (aldehyde-induced) mitochondrial dysfunction, as well as regulatory implications, and the necessary required future studies to provide scientific support for this regulation, have been covered in this review.

## 1. Aldehydes as a Component of Cigarette Smoke

The use of tobacco is the leading preventable cause of death globally, with a mortality rate of more than 8 million people per year, including 1.2 million deaths due to second-hand tobacco smoke. The most common form of tobacco use is cigarette smoking [1]. The World Health Organization (WHO) Framework Convention on Tobacco Control (FCTC) and the WHO Study Group on Tobacco Product Regulation (TobReg) address the tobacco epidemic. They focus on the prevention of initiation of tobacco use, promotion of tobacco cessation, and protection from second-hand tobacco smoke exposure. Besides these measures to reduce tobacco-related morbidity and mortality, the FCTC also acknowledges the need for the regulation of the contents and composition of emissions of tobacco products in Articles 9 and 10 of the convention, which is aimed at reducing the attractiveness, addictiveness, and toxicity of tobacco products [2,3].

The chemical composition of cigarette smoke (CS) is complex, including more than 6000 different known components [4,5]. Of these thousands of chemicals, nine toxicants are currently prioritized for mandated lowering by the WHO Study Group on TobReg. These include components in the particulate phase (e.g., specific nitrosamines and benzo(a)pyrene) and in the volatile phase (e.g., aldehydes; acetaldehyde, acrolein, formaldehyde, volatile organic compounds such as benzene and 1,3-butadiene, and carbon monoxide). These toxicants were selected based on available toxicity data, toxicity indices, their potential for mandated lowering in smoke, representativeness of the chemicals present in volatile versus particulate phases of smoke and various chemical classes, and toxicity related to health risks for diseases such as cancer, cardiovascular-, or respiratory disease [2].

In this review, we focus on the chemical class of aldehydes, mainly acetaldehyde, acrolein, and formaldehyde. These short-chain aldehydes have been identified to be formed during the pyrolysis and combustion of tobacco and, due to their chemical structure, are highly reactive electrophilic compounds. All three of these aldehydes have similar mechanisms of formation, molecular structures, and chemical properties and may be representative of the broader chemical class of aldehydes in smoke [2,6,7]. Risk assessment of these three individual aldehydes using computer models based on inhalation exposure risk factors resulted in a hazard ranking. This ranking concluded that acrolein, which is also the most studied aldehyde, represents the most harmful component of the three [8,9]. Moreover, based on computational fluid dynamics modeling coupled to airway region-specific physiologically-based pharmacokinetic tissue models, the kinetics of acrolein, acetaldehyde, and formaldehyde were analyzed for human respiratory tracts. This showed the highest local dose in oral and laryngeal tissues with penetration to the pulmonary tissues [8].

The amount of aldehydes present in CS depends on several factors, such as (added) sugars present in tobacco and smoking behavior/topography. To be more specific, the yield of aldehydes varies considerably due to the combustion and pyrolysis of sugars (e.g., caramelization) as well as the reaction between sugars and amines in tobacco [10,11,12,13]. Also, the amount and type of sugars present in tobacco fluctuate due to different drying (curing) processes of unprocessed tobacco leaves and the addition of sugars and sugar-containing ingredients during the process of manufacturing [10,14,15,16,17]. The impact of these features on the number of aldehydes per cigarette has been shown in a study by Pauwels et al. This study reported a range of 36.7–1199.5 µg acetaldehyde, 1.3–121.7 µg acrolein, and 0.7–54.9 µg formaldehyde per cigarette, with large variations depending on different tobacco brands and smoking regimes [18]. Although inhalation of tobacco smoke is a major source of aldehyde exposure in humans, it is not the only source. Also, it has been shown that aldehydes are present in emissions from e-cigarettes and thereby, as these products are gaining in popularity, contribute substantially to human exposure as well [19,20,21,22,23]. Besides smoking topography, levels of aldehydes in the emissions of these products depend on the composition of the e-liquid and the type/power of the device [24]. In general, the aldehyde levels found in e-cigarette aerosols are lower relative to tobacco smoke under normal vaping conditions, as reported in previous studies [25,26]. Kosmider et al. indicated, however, that high-voltage devices may result in increased formaldehyde and acetaldehyde levels, suggesting that product characteristics might result in a similar or even higher range of aldehydes in e-cigarette vapor compared to tobacco smoke [24]. Aldehydes are also endogenously formed, and both environmental, as well as occupational exposure to aldehydes takes place. This can occur via, for example, air pollution, burning of fossil fuels, industrial waste, and indoors via fireplaces and gas heaters/stoves. Moreover, besides inhalation, dermal or oral exposure can also be a route of exposure to aldehydes, for example, via cosmetics or diet. Exposure to these non-tobacco sources of aldehydes exposure, however, situate themselves in a low dose range compared to smoking-associated aldehyde exposure [20,27,28,29].

Aldehydes can be detoxified by various non-enzymatic and enzymatic pathways. Non-enzymatic pathways consist of cellular sulfhydryl and amine-bases nucleophiles regulating the conjugation of aldehydes. Enzymatic pathways include aldehyde dehydrogenases (ALDH) facilitating oxidation, aldo-keto reductases stimulating reduction, and glutathione inducing conjugation (in which glutathione-S-transferases are catalysators) [30]. To explain in more detail in the context of this review, an important isoform of the enzyme ALDH is the mitochondrial enzyme ALDH2. This enzyme plays a major role in detoxifying aldehydes, including acetaldehyde, acrolein, and formaldehyde, to less reactive forms [31,32,33,34]. Interestingly, although (isoforms of) ALDH enzymes are highly expressed in stem cells of all tissues, besides the liver, kidney, heart, and brain, levels of ALDH2 are observed to be expressed in stem cells of the airways and in lung tissue [35,36,37,38].

## 2. Aldehyde-Induced Respiratory Toxicity Is Associated with the Development of Chronic Obstructive Pulmonary Disease

Inhalation of aldehydes, through, for example, cigarette smoking, results in exposure of the epithelial cells lining the respiratory tract and the alveoli and has been convincingly linked to respiratory toxicity. In the following section, we discuss evidence for aldehyde-induced respiratory toxicity, specifically for acrolein, formaldehyde, and acetaldehyde individually.

With regard to acrolein, already many years ago, it was shown that sub-chronic acrolein exposure in rats (up to 4 ppm, for 62 days: 6 h/day, 5 days/week) resulted in impaired lung function and structure [39]. Due to its electrophilic nature, acrolein can react with DNA, RNA, protein, and lipids in the cells of the lungs [7,40,41]. The International Agency for Research on Cancer (IARC) has classified acrolein as ‘probably carcinogenic to humans’ (Group 2A) based on sufficient evidence of carcinogenicity in experimental animals and strong mechanistic evidence [42]. Limits of human occupational exposure vary per country: EU Occupational Exposure Limits (OEL) are 0.02 ppm (8 h time-weighted average; TWA) or 0.05 ppm (Short Term Exposure Limit; STEL) [43], and the current American Conference of Governmental Industrial Hygienists threshold limits are 0.1 ppm [44]. Moreover, a more recent mode-of-action analysis described the potential of acrolein to induce key cellular processes linked to respiratory toxicity that is associated with tobacco smoke exposure [45]. These included interaction with proteins and other macromolecules, initiation of cell death (oncosis, necrosis, and apoptosis), inflammation responses and oxidative stress, tissue remodeling, and devastation, as well as impairments in the elasticity of the lung and enlarged airspace of the lung. Interestingly, all of these processes have also been recognized to be essentially involved in the airway pathogenesis of chronic obstructive pulmonary disease (COPD) [45,46]. Moreover, acrolein-exposed mice showed elevated transcript and protein levels as well as increased activity of lung matrix metalloproteinase 9, which was associated with increased transcript levels of genes involved in mucus production (*MUC5AC*) and mucin protein in lung tissue (both features associated with COPD) [47].

Although, like acrolein, it is known that acetaldehyde and formaldehyde can induce respiratory toxicity, the underlying cellular mechanisms involved have been less extensively studied and are only scarcely described in the literature. The IARC has classified formaldehyde as ‘carcinogenic to humans’ (Group 1) based on convincing toxicological and epidemiological evidence [48] and acetaldehyde as ‘possibly carcinogenic to humans’ (Group 2B), which is based on limited human evidence and sufficient experimental animal evidence of carcinogenicity [49,50]. Formaldehyde OEL limits of inhalation are 0.3 ppm (8 h TWA) or 0.6 ppm for STEL (15 min) [51], and acetaldehyde human exposure limits are for occupational exposure 200 ppm for 8 h TWA (Occupational Safety and Health Administration) [52]. An in vitro study by Cheah et al. observed differentially-expressed genes involved in apoptosis and DNA damage after exposure of lung alveolar epithelial cells to formaldehyde or acetaldehyde [53].

In this paragraph, we discuss a few studies providing evidence for formaldehyde-induced respiratory toxicity. Regarding the impact of formaldehyde specifically, a link has been shown between formaldehyde inhalation and respiratory toxicity manifested as an inflammatory response, cytotoxicity, and genotoxicity [54]. Interestingly, a recent study investigated the individual susceptibility to lung injury due to formaldehyde exposure by integrating analyses of the genome with molecular epidemiology. A link was shown between expression of the gene 5-hydroxytryptamine (serotonin) receptor 4 (*HTR4)*, previously identified to be associated with airflow obstruction [55,56,57], and individual susceptibility to formaldehyde-induced adverse respiratory effects (progression and prevalence of COPD) [58].

With respect to acetaldehyde, a hazard summary of the US EPA described the main acute and chronic effects upon inhalation exposure [59]. Acute exposure to acetaldehyde was described to result in irritation of the respiratory tract in humans, and higher levels of exposure have been shown to result in erythema, pulmonary edema, and necrosis. Moreover, in animals, acute exposure to acetaldehyde resulted in a decline in breathing rate and increased blood pressure [60]. Although acute inhalation of acetaldehyde resulted in low toxicity in rats, rabbits, and hamsters, chronic inhalation revealed alterations in nasal mucosa and trachea in hamsters [60,61,62]. To be more specific, Kruysse et al. and Feron et al. observed histopathological changes such as necrosis, inflammation, epithelial hyper- and metaplasia in the respiratory tract (nasal cavity, larynx, trachea, and bronchi) of hamsters exposed for long-term (90-days up to 52 weeks) to high levels of acetaldehyde (1340–4560 ppm) [63,64,65]. In addition, acetaldehyde has been identified as being genotoxic and possibly carcinogenic [59,66,67], but the evidence is limited, resulting in Group 2B classification by IARC, as mentioned above. However, studies linking acetaldehyde inhalation to a potentially increased risk of developing COPD are lacking.

Besides the role of individual aldehydes in inducing cellular mechanisms underlying lung injury, a few studies also investigated the impact of a mix of reactive aldehydes, which is more representative of CS, on these processes in cells of the airways. For example, van der Toorn et al. showed a critical role for aldehydes present in CS in mediating cytokine production as well as neutrophilic airway inflammation, while mononuclear inflammation was not affected in mice exposed to CS (including aldehydes) for five days [68]. In addition, Zhang et al. have shown a synergistic impact of specific aldehydes on apoptosis, cytotoxicity, and genotoxicity using an in vitro model of bronchial epithelial cells co-exposed to acrolein and/or formaldehyde [69,70,71]. Also, Zulueta et al. exposed human bronchial epithelial cells to a mixture of nicotine (1 mM), acrolein (35 µM), formaldehyde (80 µM), and acetaldehyde (1 mM), which resulted in an increased abundance of inflammatory cytokines and *matrix metalloproteinase 9* (*MMP9*) [72]. These findings illustrate the importance of not only focusing on individual aldehydes but also taking into account the respiratory toxicity of the mixture.

To summarize, inhalation of these three short-chain aldehydes as components of CS has been convincingly associated with respiratory toxicity in which cellular mechanisms such as oxidative stress, tissue remodeling, inflammation, and cell death play a key role.

A link between (CS-related) exposure to aldehydes and the development of COPD has been suggested by several studies. Elevated acrolein levels have been observed in breath condensate and induced sputum [73] from COPD patients compared to healthy non-smoking controls, as well as in plasma from COPD patients compared to current smokers without COPD [74]. In addition, increased acrolein concentrations in supernatants of homogenized lung tissue, as well as protein-bound acrolein, have been detected in lung tissues from COPD patients and non-COPD smokers, compared to non-COPD never-smokers [74]. The fact that levels in COPD patients and smokers were found to be higher compared to never-smokers is suggestive of significant CS-related exposure. Another possibility is that somehow COPD patients or non-COPD smokers generate higher levels of endogenously-produced aldehydes. Although this, in theory, is a possibility, no studies exist to suggest this. Given the fact that CS contains significant amounts of different aldehydes and COPD patients, in general, have a substantial smoking history, it is more likely that increased levels of acrolein observed in COPD patients and non-COPD smokers originate from CS directly. Furthermore, increased expression of ALDH enzymes, including ALDH2, has been observed in bronchoalveolar lavage fluid from COPD patients [75]. Moreover, the presence of ALDH2*2 (an inactive allele of ALDH2) has been associated with an increased risk of developing smoking-associated chronic airway obstruction in a Japanese population [76]. In addition, Kuroda et al. also extensively studied the effect of this ALDH2 polymorphism on the lung using several in vivo and in vitro models. Firstly, they observed an association between the inactive form of the gene ALDH2*2 and reduced lung function parameters (FEV_1_/FVC) in a population of healthy individuals. Secondly, antioxidant gene expression was found to be decreased if *ALDH2* function was disturbed in human lungs. Lastly, conflicting results were shown in Aldh2*2 Tg mice, as they were resistant to developing emphysema in response to chronic CS exposure [77]. The susceptibility to inhalation toxicity of acetaldehyde was also studied in *Aldh2^−/−^* mice exposed for 24 h/day for 14 days and revealed more pronounced abnormalities to pathological features of the respiratory epithelium in the larynx, pharynx, trachea, and nose [78]. With respect to other ALDH enzymes, it has been shown in vitro that expression of ALDH3A1 protects against cytotoxicity and DNA damage in CS extract (CSE)-exposed epithelial cells of the airways [79].

In summary, these findings clearly indicate that smokers are highly exposed to aldehydes (most likely through CS) and that aberrations in the mechanisms responsible for detoxifying aldehydes are associated with an increased risk for abnormalities in lung function and development of obstructive airway/lung diseases such as COPD.

## 3. Mitochondrial Function in Healthy Cells of the Airways and Lungs

Interestingly, the cellular processes that have been linked to acrolein-induced respiratory toxicity and COPD development are known to be regulated by the dysfunction of mitochondria. Indeed, mitochondrial dysfunction induces cell death pathways, inflammation, and oxidative stress in multiple cell types, including airway and lung epithelial cells [80]. Healthy and functional mitochondria are important for the normal function of cells of the lungs and airways. This can be illustrated by energy generation by these organelles, which is essential for maintaining specific cellular functions, such as cilia beating and the production of surfactant and mucus [80,81]. In order to be able to respond to the energy demand of distinct cell types, the number of mitochondria and their organization is variable in different types of airway epithelial cells and alveolar cells [80,82]. Moreover, mitochondria are highly dynamic organelles capable of adapting to cellular stressors and changes in metabolic requirements [80]. Processes regulating mitochondrial turnover, mitochondrial content, and metabolism preserve the homeostasis of the mitochondrial network. These processes include the synthesis of new mitochondria (mitochondrial biogenesis) and mitochondrial dynamics, including mitochondrial fusion, mitochondrial fission, as well as targeted degradation of damaged or dysfunctional mitochondria (mitophagy). In short, mitochondrial biogenesis is primarily regulated by the coordinated action of transcriptional coactivators of the peroxisome proliferator-activated receptor gamma, coactivator 1 (PPARGC1) family and their downstream transcription factors [83,84,85,86]. On the other hand, mitophagy is controlled by two distinct pathways: the ubiquitin-mediated mitophagy pathway and the receptor-mediated mitophagy pathway. The ubiquitin-mediated mitophagy pathway is mediated by the key players PTEN-induced kinase 1 (PINK1) and parkin RBR E3 ubiquitin protein ligase (PRKN). The receptor-mediated mitophagy pathway is regulated by BCL2 interacting protein 3 (BNIP3), BNIP3-like (BNIP3L; NIX), and FUN14 domain containing 1 (FUNDC1) [86,87,88]. Mitochondrial biogenesis, fission/fusion, and mitophagy are all continuously interacting with each other to orchestrate the complex, dynamic mitochondrial network in cells of the airways and lungs [80,89].

Thus, mitochondria have an essential role in maintaining cellular homeostasis and function in epithelial cells of the airways/lungs, and mitochondrial dysfunction triggers processes known to be involved in the pathogenesis of COPD. Therefore, it comes as no surprise that mitochondrial abnormalities, including changes in the molecular mechanisms involved in mitochondrial homeostasis, have been described in these cell types in COPD, as described in more detail below.

## 4. Mitochondrial Abnormalities in Epithelial Cells of the Airways and Lungs from COPD Patients

Aberrant mitochondrial morphology has been observed in epithelial cells of the airways and lungs of COPD patients. Indeed, elongated and swollen mitochondria have been described in primary bronchial epithelial cells (PBEC) from ex-smoking COPD patients with very severe disease compared to never-smoking controls [90]. In line with these observations, another study described fragmentation, branching, and cristae depletion in PBEC from COPD patients relative to smokers without COPD [91]. Also, a reduction in mitochondrial membrane potential, as well as higher mitochondrial reactive oxygen species (ROS) and lower superoxide dismutase 2 levels (a mitochondrially-located anti-oxidant), were shown in isolated mitochondria from bronchial biopsies from COPD patients with moderate disease (GOLDII) relative to healthy never- and ex-smokers [92]. Moreover, based on the hypothesis of an association between the release of extracellular mitochondrial DNA (mtDNA) and mitochondrial dysfunction in COPD patients, mtDNA levels in urine were linked to increased respiratory symptom burden, in particular among smokers without COPD and mtDNA levels in plasma were linked to baseline COPD status [93,94]. Collectively, these studies suggest abnormalities in mitochondrial morphology/content and oxidative responses in the lungs and airways of COPD patients.

Although studies investigating the molecular regulation of mitochondrial biogenesis in the airways and lungs of COPD patients are very limited, a few studies exist in which the abundance of specific molecules involved in mitochondrial biogenesis has been assessed in PBEC or (peripheral) lung tissue from COPD patients. Reduced mRNA expression of the master regulator controlling mitochondrial biogenesis, *PPARGC1A*, was observed in lung homogenates from moderate and severe COPD patients (GOLDII and GOLDIII), whereas elevated levels of *PPARGC1A* were observed in mild COPD patients (GOLDI) [95]. In contrast to these findings, in PBEC of ex-smoking COPD GOLDIV patients, elevated transcript levels of *PPARGC1A* relative to never-smoking controls have been reported [90]. Other studies have reported decreased or no alterations in levels of TFAM in lung tissue from COPD patients compared to non-COPD patients [96] or in PBEC from ex-smoking very severe COPD (GOLDIV) patients compared to never-smoking controls, respectively [90]. Discrepancies between these studies likely relate to variations in disease severity, variability in cohorts (gender, age), or smoking history, as it has been shown that these variables can influence the expression of genes related to mitochondrial biogenesis. Also, it is important to realize that COPD is a very heterogeneous disease not only in the pulmonary compartment but also systemically, and the term ‘’COPD’’ should be considered as an umbrella term that covers multiple disease phenotypes. Disease phenotypes can range between, for example, emphysematous versus chronic bronchitis or a combination thereof, from stable disease to frequent exacerbations, and also different inflammatory patterns could be recognized (neutrophilic versus eosinophilic) [97,98]. Taking this into consideration, it is likely that these variables contribute to the seemingly contradictory findings in relation to the molecular regulation of mitochondrial biogenesis (e.g., PPARGC1A and TFAM) described above. Collectively, although scarce in number, these studies, however, do suggest that the molecular regulation of mitochondrial biogenesis can be altered in the airways and lungs of COPD patients.

With respect to the regulation of mitophagy in the lungs/airways in COPD, an increased expression of PINK1, involved in ubiquitin-mediated mitophagy, has been observed in lung tissue homogenates and in bronchial epithelial cells from COPD patients [90,99]. On the other hand, decreased protein levels of PRKN, another important regulator associated with the ubiquitin-mediated mitophagy pathway, were found in lung homogenates from COPD patients relative to non-COPD smokers [100]. This decrease in PRKN protein levels was also demonstrated in isolated mitochondria from lung tissue from COPD patients or smokers relative to non-smokers, suggestive of an altered translocation of PRKN to the mitochondria in COPD. These findings may indicate impairments in the clearance of (damaged) mitochondria through the mitophagy pathway [101]. Research assessing the molecular regulation of the receptor-mediated pathway in cells of the airways and lungs from COPD patients is scarce. Besides the observed changes in the regulation of mitophagy in COPD patients, also upregulated expression of autophagy proteins, as well as the increased formation of autophagic vacuoles, has been observed in lung tissue from COPD patients compared to non-smokers, respectively elevated at GOLD0 and sustained during disease progression up to GOLDII-IV [102].

Altogether, although not abundant in nature, these reports all suggest aberrations in the molecular pathways involved in mitochondrial content, mitochondrial function, and mitochondrial quality control (mitochondrial biogenesis versus mitophagy) in cells of the airways in COPD.

## 5. CS-Induced Mitochondrial Dysfunction in Epithelial Cells of the Airways and Lungs

Given that smoking is the main risk factor for developing COPD [103] and, as described above, several abnormalities at the level of the mitochondrion have been described in COPD patients, it is reasonable to assume a role for (components) of CS in these mitochondrial abnormalities. Indeed, multiple reviews have discussed studies that show a direct link between exposure of airway/lung epithelial cells to CS and disturbances in (the molecular regulation of) mitochondrial function both in vivo and in vitro [82,104,105]. Key findings related to CS-induced alterations in mitochondrial morphology and processes involved in maintaining mitochondrial homeostasis are described below. It has to be noted that exposure models found in the literature vary between CS, CSE, or whole CS (WCS), which obviously can vary in aldehyde content. CS and WCS are interchangeable denominations and contain both gaseous and particulate mainstream CS components, including aldehydes. Levels of aldehydes in (W)CS are variable and depend on brand and smoking regime/topography [18]. CSE, on the other hand, by nature contains only water-soluble CS components. It is reasonable to assume that, due to the volatile nature of aldehydes, levels of aldehydes in aqueous extracts like CSE likely are low.

Firstly, abnormalities in the mitochondrial structure (e.g., fragmentation) have been convincingly demonstrated upon CSE exposure of murine alveolar cells and human small airway- and bronchial epithelial cells [90,91,106,107], which is similar to observations in COPD patients as described in the paragraph above.

Moreover, alterations in mitochondrial bioenergetic processes in response to CS(E) exposure have been reported in several airway models. More specifically, decreased mitochondrial respiration, lower ATP production, elevated mitochondrial ROS levels, damaged mtDNA, altered abundance/activity of subunits of electron transport chain complexes, and loss of mitochondrial membrane potential have been observed in vitro in alveolar- and bronchial epithelial cells (primary cells or cell lines) in response to CSE [90,91,99,106,108,109,110,111]. In addition to these in vitro studies, decreased mitochondrial respiration and lower ATP production while metabolizing glucose, altered glycolysis affecting the surfactant synthesis, and increased expression/activity of (subunits of) oxidative phosphorylation complexes have been observed in isolated primary alveolar type II cells or lung tissue from mice exposed to CS for four or eight weeks [112,113]. Conflicting findings suggesting an adaptive, recovery or compensatory response have also been reported in primary alveolar type II cells upon low, non-toxic doses of smoke or following eight weeks of smoke exposure in mice followed by recovery [107,113].

In line with the CS-induced abnormalities in mitochondrial morphology and function, studies have also shown that CS can impede the molecular pathways involved in the control of mitochondrial quality processes. Indeed, both in vitro and in vivo CS exposure models convincingly showed alterations in the abundance of key molecules involved in these pathways.

More specifically, upregulated transcript levels of key regulators of the PPARGC1-network, respectively *PPARGC1A* and *PPRC1*, have been shown in bronchial epithelial cells (in basal fraction) acutely exposed to CS or extracts thereof, whereas decreased gene expression of *PPARGC1B* has been reported [114,115]. In contrast, a decrease in the expression of PPARGC1A has been observed in experimental animal models of CS-induced COPD, e.g., in mice instilled with CSE [116] or in mice instilled with LPS and CS [117].

With regard to mitophagy, in line with the observation in COPD patients, an increased abundance of PINK1 and decreased abundance of PRKN was observed in various in vivo and in vitro airway models of CS(E) exposure [99,101,108,114,118,119]. Conflicting findings or no alterations in the abundance of these constituents were also reported in human or mice alveolar or bronchial epithelial cells exposed to CSE [90,99,101,106,107,108]. The other mitophagy pathway, i.e., receptor-mediated mitophagy, was less well studied before, and contradictory results were reported. An elevated abundance of receptor-mediated proteins has been observed both in vivo in a COPD mouse model (exposed to CS) and in vitro in bronchial epithelial cells following CSE or WCS exposure [114,120,121]. Interestingly, a leading role for CS-induced receptor-mediated mitophagy in COPD has been suggested by Wen et al., showing suppression of the development of COPD-like features in mice by silencing *FUNDC1* [121].

Whether or not CS-induced changes in mitophagy and autophagy in the airways and lungs can be considered as being protective or detrimental in the context of lung disease remains to be established. Illustrative of this complex regulation of mitophagy is the fact that in vivo and in vitro studies reported a protective role of both knockdown/knockout of *PINK1* or *PRKN* [99,100] as well as of overexpression of *PRKN* [100,101,122] against CS or extract thereof induced mitophagy or dysfunctional mitochondria in cells of the lungs and airways. Future research is required to elucidate the exact role of these key regulators herein.

Mitochondrial fusion and fission are essential processes involved in the biogenesis of new organelles and in the degradation of defective mitochondria. In line with CS-induced abnormalities in these processes, CS has been shown to disrupt fusion and fission events. Indeed, CS(E) exposure has been shown to lead to mitochondrial fragmentation (increased fission proteins) and decreased fusion (proteins) in epithelial cells of the airways, as shown in previous studies [91,99,106,118,119,123,124]. Nevertheless, conflicting findings pointing towards a decreased abundance of fission- and increased abundance of fusion-associated proteins or even no alterations are reported upon CSE or WCS (condensate) exposure in cells of the lungs and airways [90,101,107,114,125]. Possible explanations for these discrepancies could be the variation in models and duration of exposure illustrated by Walczak et al. [126], cell-type-specific effects, and the fact that fission and fusion are dynamic processes operating as a flux. Further studies should investigate the functional impact of this disbalance.

Although the body of evidence described above clearly shows (CS-induced) abnormalities at the levels of the mitochondrion and the pathways regulating mitochondrial content and function in COPD, these studies do not provide evidence for a causal relationship between mitochondrial dysfunction in epithelial cells of the airways and lungs and the development of COPD. One study by Cloonan et al., however, did provide causal evidence for this by demonstrating that prevention of CS-induced mitochondrial dysfunction in a mouse model of COPD significantly ameliorated CS-induced development of emphysema and bronchitis [127]. This study further highlights the central role of mitochondria in the development of COPD.

Thus far, it is unknown which exact chemical components present in CS are responsible for mitochondrial dysfunction. Aldehydes are suggested to be one of the chemicals involved, as outlined below.

## 6. Aldehydes-Induced Mitochondrial Dysfunction in Lung Cells Associated with COPD Pathogenesis

In this section, we describe evidence from in vitro and in vivo studies that suggest that aldehydes, as components of CS, may mediate CS-induced mitochondrial abnormalities. As this is a relatively new vantage point, literature regarding this topic is limited and mainly centers around investigations of the impact of acrolein on mitochondrial metabolism in vivo and in vitro.

Abnormal mitochondrial morphology (e.g., fragmentation) has been shown upon exposure of human lung fibroblasts and alveolar epithelial cells to acrolein [128]. Moreover, *Aldh2^−/−^* mice, deficient in aldehyde detoxification mechanisms, have been shown to display aberrant mitochondrial morphology as well as elevated ROS levels and less functional mitochondria in cells of the trachea and lung compared to WT mice, even in the absence of exposure to CS [77].

Previous studies also described a negative impact of acrolein on the regulation of mitochondrial function and energy metabolism in the airways and lungs. For example, in an in vivo study in which rats were acutely (nose-only) exposed to 4 ppm acrolein, a shift towards a more glycolytic metabolism was observed in the lung, indicated by increased lactate levels and increased abundance of key glycolytic enzymes [129]. Moreover, in similar animals from the rat study mentioned above, reduced mRNA and protein levels of subunits of electron transport chain complexes were found in the lungs upon acute 4 ppm acrolein exposure [129]. Another study using 10 ppm acrolein exposure revealed increased molecules associated with glycolysis and branched-chain amino acid metabolism in acrolein-sensitive and acrolein-resistant mouse strains by metabolic profiling [130]. Besides this in vivo evidence, in vitro exposure of alveolar epithelial cells to acrolein resulted in a metabolic shift, respectively, from the glycolytic pathway to the pentose phosphate pathway in mitochondria. This shift results in the use of palmitate from phosphatidylcholine as an alternative substrate for mitochondrial respiration which may lead to decreased surfactant levels, respectively, a feature often observed in lung diseases including COPD [131]. In addition, acrolein exposure to alveolar lung cells was shown to induce cellular stress and apoptosis, which was mediated through the mitochondrial pathway controlled by ROS [132]. Furthermore, mtDNA damage, reduced mitochondrial membrane potential, inhibited bioenergetics (decrease in mitochondrial respiration), and decreased mtDNA copy number have been observed in alveolar epithelial cells and fibroblasts following acute acrolein exposure [128]. Lastly, exposure of human lung fibroblasts to low doses of acrolein for a prolonged period of 16 days resulted in significantly decreased mitochondrial membrane potential and downregulated expression of complex I, II, III, and V of the mitochondrial electron transport chain [133]. In summary, these findings show that acrolein exposure can disrupt the regulation of mitochondrial function and mitochondrial metabolism in cells of the airways and lungs.

Additional knowledge about the potential of aldehydes to disrupt mitochondrial metabolism and bioenergetic processes can be obtained from studies using cell types other than lung cells. In this regard, acrolein incubation of mitochondria isolated from rat liver resulted in dose-dependent inhibition of mitochondrial respiration, mitochondrial enzyme activity associated with complex I and II, pyruvate dehydrogenase, alpha-ketoglutarate dehydrogenase, as well as alteration of mitochondrial permeability transition and elevated protein carbonyls [134].

Knowledge regarding the effect of acetaldehyde and formaldehyde on the regulation of mitochondrial metabolism in the airways is limited and mainly studied in non-lung cell types. One exception is the reported impaired cilia function and cilia beating in bovine bronchial epithelial cells upon acetaldehyde exposure implying disrupted mitochondrial energy metabolism [135,136]. To explain in more detail the findings in other cell types, dysregulated mitochondrial metabolism has been observed in acetaldehyde-exposed hepatocytes [137], upon formaldehyde and acetaldehyde exposure of rat liver mitochondria [138] and after formaldehyde exposure of neuroblastoma cells [139]. Moreover, in macrophages, formaldehyde exposure resulted in increased glycolysis and hypoxia signaling [140].

In addition to acrolein, acetaldehyde, and formaldehyde, a few studies assessed the impact of crotonaldehyde (which is also found in CS) on mitochondria in cells of the airways. Recently, in vivo chronic exposure of rats to crotonaldehyde by oral gavage induced apoptosis in lung tissue which was associated with inflammation and oxidative stress, mediated via the mitochondrial pathways [141]. Chronic crotonaldehyde exposure in rats by intragastric administration also resulted in mitochondrial dysfunction in the lungs, evidenced by disrupted mitochondrial energy metabolism, oxidative stress, and apoptosis [142]. In addition, in vitro studies reported crotonaldehyde-triggered apoptosis (caspase-dependent manner) as well as oxidative stress in human bronchial epithelial cells [143] and showed crotonaldehyde-induced apoptosis via intracellular calcium, mitochondria, and P53 signaling in alveolar macrophages [144]. In other cell types, it has been shown that short-term crotonaldehyde exposure of mice cardiomyocytes resulted in apoptosis, mitochondrial injury, and inhibited regulatory pathways involved in mitochondrial oxidative capacity [145].

Interestingly, Clapp et al. demonstrated that dose-dependent exposure to cinnamaldehyde, yet another member of the aldehyde family of chemicals (present in e-cigarette liquids), impaired mitochondrial function, mitochondrial respiration, glycolysis, and reduced intracellular ATP levels and suppressed ciliary beat frequency of differentiated human bronchial epithelial cells [146].

Although these studies clearly suggest a detrimental impact of different aldehydes, commonly associated with CS, on mitochondrial function and mitochondrial metabolism, limited information is available on whether or not aldehydes disturb the regulation of mitochondrial biogenesis and mitophagy. We recently observed decreased expression of PPARGC1A and its downstream transcription factors involved in mitochondrial biogenesis one day after acrolein exposure in rats. After two days of exposure, however, upregulated transcript levels of *PPARGC1B* were observed in lung homogenates of these animals, suggestive of a compensatory response [129]. In line with this, a decline in PPARGC1A protein levels has been reported in vitro in lung fibroblasts upon acrolein exposure [133]. In addition, reduced PPARGC1A protein levels were also observed in mice cardiomyocytes upon short-term crotonaldehyde exposure [145]. To the best of our knowledge, there are no previous studies available investigating the effect of acetaldehyde and formaldehyde on the molecular regulation of mitochondrial biogenesis in cells of the airways or lungs.

With respect to mitophagy, it has been shown in the literature that aldehydes can induce autophagy and mitophagy, which is mainly studied in response to acrolein in non-primary human lung cells. In vivo studies reported minimal changes in the abundance of markers associated with mitophagy and autophagy in lung homogenates of rats nose-only exposed to acrolein [129] and augmented autophagosome number (and protein ratio MAP1LC3BII/I) in alveolar epithelial cells from rats upon sub-acute exposure to formaldehyde [147]. Moreover, acrolein-induced autophagy and mitophagy have been demonstrated in human lung alveolar basal epithelial cells and fibroblasts [128].

In addition, the impact of acrolein on mitochondrial dynamics has been evaluated previously to a limited extent. Acrolein exposure altered transcriptional regulation of mitochondrial fission and fusion in vivo (in rat lung homogenates) and induced mitochondrial fission in vitro (in alveolar and lung fibroblasts) [128,129].

**Figure 1:**
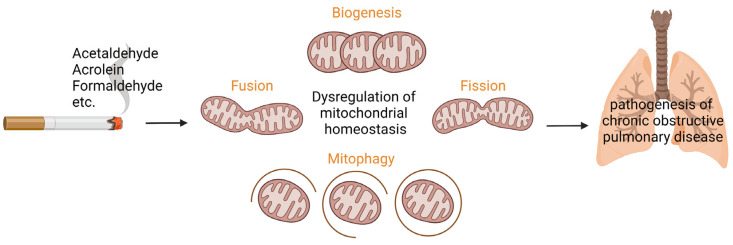
Aldehydes present in cigarette smoke are suggested to induce dysregulation of processes controlling mitochondrial homeostasis in cells of the airways and lungs, respectively the synthesis of new mitochondria (mitochondrial biogenesis) and mitochondrial dynamics including mitochondrial fusion/fission as well as targeted degradation of damaged or dysfunctional mitochondria (mitophagy). Based on previous (limited) in vitro and in vivo studies, aldehydes-induced mitochondrial dysfunction has been suggested to be involved in the pathogenesis of chronic obstructive pulmonary disease. Designed with Biorender.com.

To summarize, to our current knowledge, exposure to smoking-associated aldehydes, in particular, acrolein, results in dysregulated mitochondrial (energy) metabolism, mitochondrial biogenesis, and mitophagy in cells of the airways and lungs (Figure 1). It is, however, difficult to compare the abovementioned findings in the distinct studies because of differences in experimental set-ups, such as exposure dose, exposure regime, and types of cells analyzed (lung versus non-lung cells).

## 7. Potential Therapeutical Applications Targeting Aldehyde-Induced Mitochondrial Dysfunction in COPD

### 7.1. Mitochondria as Therapeutic Target in COPD

Emerging evidence describes the potential of mitochondrial-targeted therapy aimed at improving mitochondrial function or rescue/reverse mitochondrial dysfunction to protect against the development of (CS-induced) COPD and/or optimizing lung function. As summarized in previous reviews, these therapeutical applications target mitochondrial transfer (stem cells), inhibition of mitophagy (Quercetogetin), mitochondrial dynamics (Drp1 inhibitors: Mdivi-1, P110), mitochondrial ROS scavengers/antioxidants (e.g., MitoTEMPO, MitoQ, Tiron, SS-31, N-acetylcysteine), iron chelators (Deferiprone), stimulation of mitochondrial biogenesis (AMPK activators, SIRT1 overexpression/activation), and mitochondrial therapy (mitochondrial transplantation/mitoception) [82,148,149]. Other mitochondrial-targeted therapies focus on for example ameliorating mitochondrial respiration by targeting the reprogramming of mitochondria influenced by lifestyle, modification of let-7/lin28axis, and miRNAs: hypoxamirs or mitomirs. Moreover, a new direction in this field focuses on the crosstalk between microbes and mitochondria, e.g., microbial metabolites which are sources for mitochondrial oxidation, altered fatty acids affecting mitochondrial function, and stimulators of mitochondrial biogenesis, for example, pyrroloquinoline quinone [150].

### 7.2. Targeting Aldehydes

As it can be suggested, based on the abovementioned literature, that aldehydes are implicated in the pathogenesis of COPD via dysregulating mitochondrial function, neutralizing/detoxifying aldehydes might be a potential approach for COPD therapy. Compared to the studies mentioned above, which focus on targeting mitochondria, fewer studies focus on specifically targeting aldehydes metabolism. The studies available, in particular, investigate therapeutic targets in relation to ALDH2 and exposure to acrolein.

Firstly, some studies target the enzyme ALDH2 using alda-1, an agonist and structural chaperone of the mitochondrial isoform of ALDH. It has been shown that alda-1 treatment resulted in elevated ALDH2 activity, respectively ALDH2*1/*1 (WT) activity 2.1 fold, ALDH2*2 11 fold, and homozygotes/heterozygotes of the allele 2.2 fold [151]. The working mechanism by which alda-1 mediates this is by binding at the entrance of the catalytic domain and allosteric activation of ALDH2. In the case of ALDH2*1, binding of alda-1 resulted in elevated efficiency of acyl-enzyme hydrolysis, and in the case of ALDH2*2 structural stability is restored [152]. This allosteric activation of ALDH2 has been linked to enhanced clearance of aldehydes and subsequently reduced levels of aldehydes [151,153,154,155]. Alda-1 as a therapeutic target was mainly investigated in the context of acute lung injury. For example, it has been shown that administration of alda-1 protected against acrolein-induced lung injury, which was manifested as a reduction in edema and inflammation in mice. Moreover, in vitro exposure to alda-1 mitigated the acrolein-induced increase in endothelial monolayer permeability in rat lung microvascular endothelial cells, while inhibition of mitochondrial respiration upon acrolein exposure was not prevented by alda-1 treatment [156]. In recent studies, it was demonstrated that alda-1 treatment prior to hypoxia exposure alleviated mitochondrial dysfunction in lung microvascular endothelial cells [157], and continuous delivery of alda-1 attenuated the development of hypoxia-induced lung injury in mice [158]. In addition, it has been shown that alda-1 treatment protected against whole-body heating-induced acute lung injury in mice [159], and alda-1 also reduced pyroptosis and ferroptosis, processes involved in sepsis-induced acute lung injury [160]. Lastly, alda-1 might suppress lung tumor progression [161], and alda-1 has been shown to trigger the clearance of reactive aldehydes resulting in the prevention of pulmonary epithelial barrier dysfunction upon severe hemorrhagic shock [162]. Albeit in other cell types than lung cells, a few studies investigated and reviewed the potential of alda-1 as a therapeutic target in non-lung injury-related diseases by showing their attenuating impact on hepatic fibrosis and steatosis, atherosclerosis, heart failure, cardiomyopathy, and ischemia-reperfusion in several organs including heart, brain, and kidney [151,163,164,165,166]. Summarized, there seems to be therapeutic potential for alda-1 in the context of aldehydes-induced mitochondrial dysfunction, but additional fundamental and clinical research is required to investigate the potential of this agonist.

Secondly, aldehyde scavengers might be an effective therapeutic target of which, especially acrolein scavengers, have been studied. Nitrogen-containing carbonyl compounds are capable of scavenging aldehydes and are known to counteract acrolein-induced protein carbonylation [46]. Examples of these compounds are anti-hypertensive hydralazine [167], methoxyamine [168] and glycoxidation inhibitors, aminoguanidine, and carnosine [169]. Another compound suggested to have acrolein-scavenging properties is erdosteine [46]. This is an antioxidant, respectively a thiol-containing molecule, and is already used in the treatment of COPD as a mucolytic agent [170]. In more detail, in a clinical study using long-term erdosteine treatment in COPD patients, it was shown that the use of erdosteine has clinical benefits, such as fewer exacerbations and shorter hospitalization time, no reduction in lung function and an improved quality of life (health-related) [171]. Moreover, it has been shown that the use of erdosteine in stable COPD patients resulted in a reduction of oxidative stress and inflammation markers, respectively, ROS levels in peripheral blood and proinflammatory cytokine levels in bronchial sections as well as reduced eicosanoids levels [172,173,174]. Another compound, dimercaprol, has been described as an acrolein scavenger in in vitro and animal studies focusing on neural diseases. To be more specific, dimercaprol is reported to be effective in detoxifying acrolein in vitro by prevention of acrolein-induced cell death in a dose-dependent manner in PC-12 cells, and in vivo reduced acrolein levels were detected in case of spinal cord contusion injury in rat [175]. Moreover, dimercaprol provides neuroprotection in vivo in a rat model of Parkinson’s disease [176]. Formaldehyde- or acetaldehyde-scavengers are less well studied; however, metformin is suggested to be a potential formaldehyde scavenger in HepG2 cells [177].

In summary, extensive further research is required to investigate the potential application of therapeutic strategies specifically targeting mitochondria and/or aldehydes in the context of lung disease; in particular, the clinical potential of alda-1 and acrolein scavengers has to be assessed.

## 8. Regulation of Aldehydes in CS

In this paragraph, we will discuss the regulatory implications in tobacco control and knowledge gaps for additional research in the niche of aldehydes-induced mitochondrial dysfunction in the context of COPD pathogenesis.

### 8.1. Implications for Regulation

The scientific evidence discussed in this review describes the available support for the regulation of aldehydes in CS. For this, we focused on the mechanistic and causal impact of aldehydes, in particular acrolein, on dysregulated mitochondrial (energy) metabolism, mitochondrial biogenesis, and mitophagy in epithelial cells of the airways and lungs. Besides strategies to prevent aldehydes-induced damage (e.g., aldehydes-scavenger), also aldehyde content can be reduced in cigarettes to protect smokers from high aldehyde exposure levels. Reduction of aldehyde content in cigarettes can be achieved by decreasing sugar content, selecting specific tobacco types, and cigarette design (e.g., cigarette filter ventilation) [10,18,178]. Moreover, smoking behavior and topography are known to influence the number of aldehydes a smoker is exposed to [179].

Additional research is required to prove if and to what level a reduction of specific aldehydes per cigarette is required to have a less harmful effect in relation to mitochondrial (dys)function and COPD development. A few studies investigated the dose effect of acrolein in vitro, showing the differential cytotoxic impact of a high dose of acrolein (cytotoxic) versus a low dose of acrolein (no cytotoxicity) in human bronchial epithelial cells [180]. In the case of acrolein, this dose-dependent effect has also been shown with respect to the immunological response. This immunological response to acrolein exposure is dependent on the dose and cell type used. Acute high doses of acrolein are suggested to result in an inhibition of the innate immune and inflammatory responses resulting in increased susceptibility to infections, and in contrast, chronic low doses of acrolein may lead to inflammation, oxidative stress, or a response leading to tissue injury [45,181]. In addition, a dose-dependent impact of acrolein exposure on the regulation of mitochondrial quality control processes and mitochondrial metabolism (e.g., membrane potential, ATP content) has been shown in vivo (0–2–4 ppm acrolein for one or two days) [129] and in vitro (0–100 µM) [128] in cells of the airways. These studies did, however, not take into account physiologically-relevant exposure regimes, as smokers are normally exposed to peak-exposure levels (puff regime) several times (i.e., cigarettes) a day for several years. In conclusion, future realistic dose-response studies should take into account a physiologically relevant exposure regime to investigate the risks and potential benefits of reducing aldehydes per puff in the context of mitochondrial (dys)function and the pathogenesis of COPD. Lastly, it should be stressed that acrolein, acetaldehyde, and formaldehyde are three of the nine chemicals prioritized by the WHO for regulation but also only represent three of the 6000 potential detrimental chemicals present in CS. Indeed, CS is a complex mixture of chemicals of which many have been shown to be harmful for human health. Further research into those other components as well as into their mixture effect should be conducted. Moreover, we would like to stress that a ‘healthy’ cigarette does not exist and cannot be designed by reducing aldehydes, but less toxic cigarettes might be less harmful for those who are not able to quit smoking.

### 8.2. Implications for Future Research

Several limitations of experimental approaches used in the studies discussed should be overcome in the future.

The current literature focuses mainly on assessing the impact of acute exposure of aldehydes in in vitro cell line models or in vivo animal models. Moreover, primarily acrolein has been studied, which is only one of the aldehydes present in CS. Future research should study, in more detail, the exposure of primary cells that more realistically mimic the human lung and the impact of other aldehydes, including formaldehyde, acetaldehyde, crotonaldehyde, and cinnamaldehyde. In addition, airborne exposure and smoking topography (puff-like manner exposure) is not taken into account in most of these studies, as well as chronic exposure is underrepresented in the available scientific evidence, as discussed above.

Besides the impact of individual aldehydes, the effects of mixtures of aldehydes present in CS should be investigated more extensively in the future. The interaction of aldehydes in a mixture might induce additive or synergistic toxicity, which could be suggested based on their common mechanisms [7]. It is important to address the potential additive or synergistic toxicity not only in the combination of pure aldehydes in a mixture but also in the aldehydes of interest combined with any of the other 6000 chemicals, including other aldehydes present in CS. This is important because of the suggestion that a mixture of pure aldehydes will behave differently from the same quantity of aldehydes present in CS, which can be explained by the condensation of aldehydes on aerosol particles in CS. Moreover, other constituents present in CS than these three aldehydes (e.g., nitrosamines, carbon monoxide, nicotine, polycyclic aromatic hydrocarbons) could play a role in the mitotoxicity of CS on which data is lacking, and additional research should be conducted. For example, it has been shown that exposure of nitrosamine NNK, present in CS, to human A549 cells, results in elevated levels of ALDH-positive cells [182].

Moreover, it should be considered that COPD development in animal models is not reflective of COPD in human patients. As chronic exposure to CS (six months) will result in mild emphysema and pulmonary lesions in small rodents, this is not comparable to GOLDIII and GOLDIV status [183,184,185]. This makes it challenging to investigate new (pharmacological) targets of COPD in in vivo experimental models and support the need for developing innovative experimental models using, for example, co-culture models including other cell types than epithelial cells, e.g., macrophages or fibroblasts, which has been shown to be promising [186,187].

In conclusion, from the evidence provided in this review, it can be concluded that aldehydes, as components of CS, are capable of interfering with the molecular regulation of mitochondrial function and metabolism in cells of the airways and lungs which may predispose to the development of COPD. Future research is necessary to reveal the underlying molecular mechanisms and causal relationships between aldehydes, mitochondrial dysfunction, and the airway pathogenesis of COPD, which will contribute to providing insights into novel therapeutic modalities aimed at aldehydes or mitochondria.

## Data Availability

Not applicable.
